# Drugging the epigenome in the age of precision medicine

**DOI:** 10.1186/s13148-022-01419-z

**Published:** 2023-01-11

**Authors:** Taylor Feehley, Charles W. O’Donnell, John Mendlein, Mahesh Karande, Thomas McCauley

**Affiliations:** 1 Omega Therapeutics, 20 Acorn Park Drive, Suite 400, Cambridge, MA 02140 USA; 2 Flagship Pioneering, 55 Cambridge Parkway Suite 800E, Cambridge, MA 02142 USA

**Keywords:** Epigenetics, Epigenomics, Precision, Specificity, Therapeutics

## Abstract

**Background:**

Modulating the epigenome has long been considered a potential opportunity for therapeutic intervention in numerous disease areas with several approved therapies marketed, primarily for cancer. Despite the overall promise of early approaches, however, these drugs have been plagued by poor pharmacokinetic and safety/tolerability profiles due in large part to off-target effects and a lack of specificity.

**Results:**

Recently, there has been marked progress in the field on a new generation of epigenomic therapies which address these challenges directly by targeting defined loci with highly precise, durable, and tunable approaches. Here, we review the promise and pitfalls of epigenetic drug development to date and provide an outlook on recent advances and their promise for future therapeutic applications.

**Conclusions:**

Novel therapeutic modalities leveraging epigenetics and epigenomics with increased precision are well positioned to advance the field and treat patients across disease areas in the coming years.

## Introduction to epigenetics

**Fig. 1:**
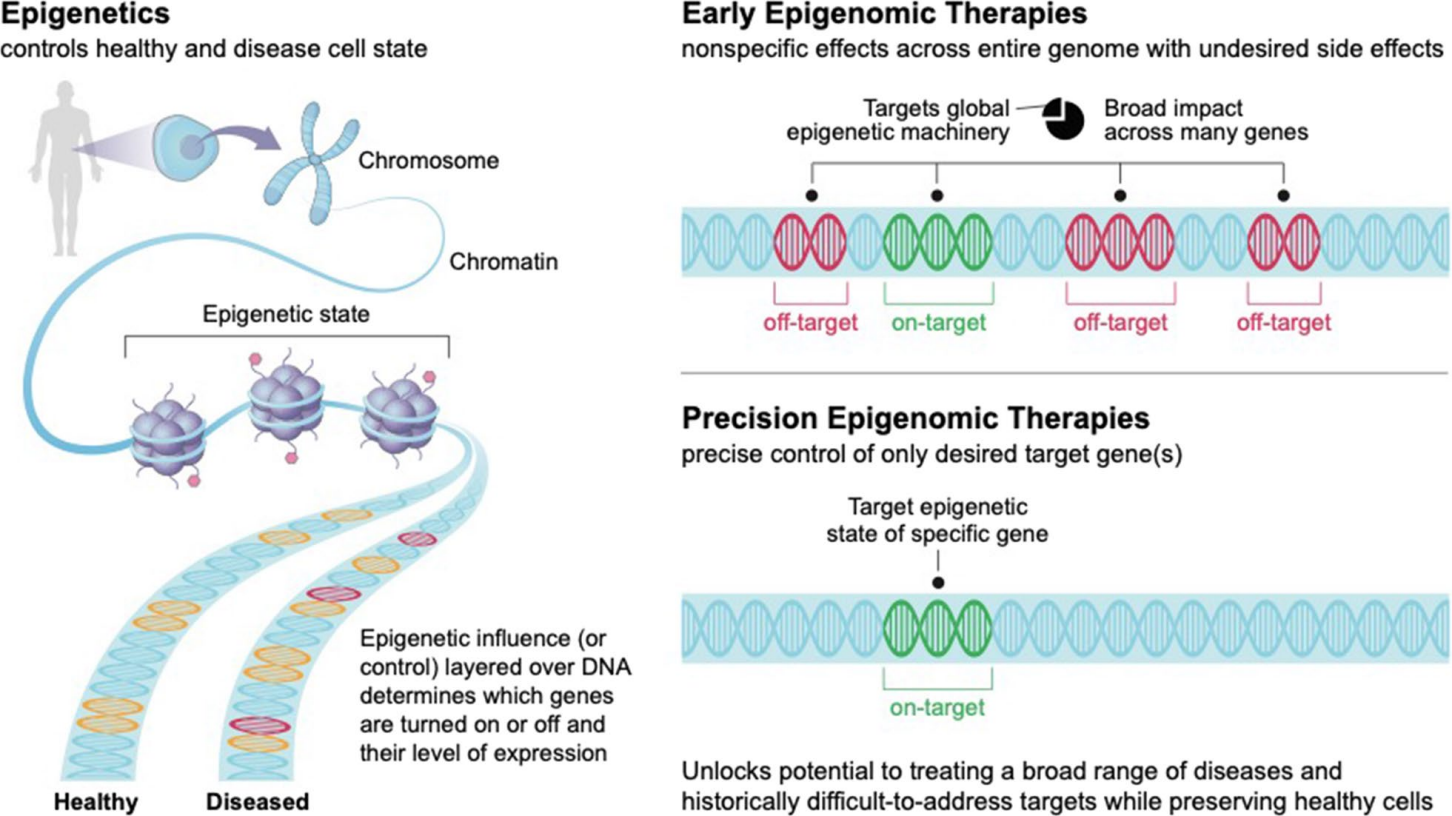
Precision epigenomic therapies have the potential to improve efficacy and tolerability. Early epigenomic therapies are limited by a lack of specificity, leading to off-target effects and more narrow therapeutic utility, as well as more limited tunability and durability. Precision therapies that act at discrete, specific loci should mitigate these challenges while delivering on the therapeutic promise of epigenomic modulation

In the 70 years since the term “epigenetics” was first coined, the field has yet to fulfill its true therapeutic potential, but has nonetheless proven a boon to basic researchers, to understand how cells process genetic information, differentiate, and respond to external stimuli [1]. At its core, epigenetics is focused on how cells control gene activity without changing the DNA sequence. This involves the modification of chemical signatures on DNA and its structures to alter the means by which transcription factors and other machinery interpret genetic information to control gene expression. Epigenetic modifications can induce changes in the accessibility of DNA as it is wound around histones, cause regulatory sequences to become refractory or amenable to transcription factor binding, or drive compartmentalization to activate or inactivate whole genomic loci. This complex system has been referred to as the “epigenetic code,” [2, 3] and describes the fundamental information layer that cells rely on to integrate and process the impact of external stimuli, in the context of past stimuli and cell-type determination despite a fixed genetic sequence (Fig. 1). With the advent of high-throughput sequencing and methodologies to interrogate chromatin state and DNA/RNA/protein interactions, an integrated understanding of epigenetics, functional genomics, and chromatin biology has blossomed into the current field of epigenomics.

Epigenetic effectors, enzymes capable of inducing changes in chromatin state, are varied and are often divided into three major categories—writers, erasers, and readers [1]. At the most basic level, writers create new epigenetic marks while erasers eliminate epigenetic marks. Finally, readers interpret the marks to change the conformation of DNA or histones and recruit additional machinery when needed.

### The promise of epigenetic therapies

The principal promise of epigenetic-based therapies is the ability to control gene expression directly at the pre-transcriptional level and thus correct gene dysregulation at its source. Perhaps the greatest benefit of this approach lies in being able to turn gene expression up or down in a durable but (typically) not permanent manner, without making any changes to the underlying genomic sequence. This capability aids in the study of cellular differentiation, lineage specification, and programming as well as enabling this understanding to be harnessed to treat disease.

The ability to leverage the endogenous mechanisms by which cells control gene expression seemed like a new key to unlock therapeutic avenues for a variety of diseases. Cancer has been, and remains, an exemplar for the utility of epigenetic modulation as a therapeutic approach [4]. In many cancers, critical tumor suppressor genes are deactivated by hypermethylation or oncogenes are activated by demethylation, leading to dysregulated gene expression and unchecked growth. Oncology is not the only therapeutic area that could benefit from such intervention, however, as a number of inflammatory and neurological disorders, as well as rare monogenic conditions, degenerative diseases and diseases of aging, have also been shown to be linked to epigenetic dysregulation [5–7]. In fact, most diseases, irrespective of etiology, occur due to gene dysregulation and should be amenable to corrections. Being able to correct these defects represents a vast opportunity to improve patient outcomes in a variety of indications.

Unfortunately, translating these advances in our understanding of epigenetics into medicines has proven more challenging than initially anticipated. While there are eight FDA-approved and marketed epigenetic therapies with six to treat hematologic malignancies and two approved for use in solid tumors (Table 1), trials of current epigenetic therapies have shown greater toxicity than expected, likely due to low specificity. Even in cases where there is activity, toxicity driven by the broad impact of global inhibition of these effectors, due to lack of cell-type and genomic specificity, can drastically limit the utility of these compounds; global changes in methylation and acetylation patterns and/or interference in large, macromolecular complexes can have unintended consequences. The relatively greater success in hematologic cancers may also be related to inherently higher sensitivity of hematopoietic lineages to epigenetic modulations relative to other cell types due to greater plasticity of cellular programs, allowing for efficacy with a narrower therapeutic window or at lower and less toxic doses. Increasing the specificity of epigenetic approaches, at both the cellular and molecular levels, as well as their durability could help bridge the gap between the promise of these therapies and the current realities of bench-to-bedside translation.

**Table 1:** Summary of epigenetic approaches and molecules

Epigenetic effector class	Drug	Modality	Marketed/developed by	Status/uses
1st generation DNA methyltransferase inhibitor	5-Azacytidine (Onureg, Vidaza)	Small molecule	Bristol Myers Squibb	FDA approved for the treatment of myelodysplastic syndrome
	5-Aza-2′-deoxycytidine (decitabine; Inqovi)	Small molecule	Astex/Taiho	FDA approved for the treatment of myelodysplastic syndrome
	Pseudoisocytidine	Small molecule	Various	Clinical development discontinued for hepatotoxicity concerns
	5,6-Dihydro-5-azacytidine (DHAC)	Small molecule	Various	Clinical development discontinued for cardiotoxicity concerns
2nd generation DNA methyltransferase inhibitor	Guadecitabine (SGI-110)	Small molecule	Astex Pharmaceuticals	Development discontinued due to lack of Phase 3 efficacy
	Fluorocyclopentenylcytosine (RX-3117, TV-1360)	Small molecule	Rexhan Pharmaceuticals (Ocuphire Pharma)	Clinical development paused due to weak Phase 2a data
1st generation histone deacetylase inhibitors	Suberoylanilide hydroxamic acid (SAHA, vorinostat, Zolinza)	Small molecule	Merck	FDA approved for the treatment of cutaneous T cell lymphoma
	Romidepsin (Istodax)	Small molecule	Bristol Myers Squibb	FDA approved for the treatment of cutaneous T cell lymphoma; accelerated approval for peripheral T cell lymphoma withdrawn
	Sodium butyrate/butyric acid	Small molecule	Various	Research compound to explore HDAC inhibition in model systems
2nd generation histone deacetylase inhibitors	Belinostat (Beleodaq)	Small molecule	Acrotech Biopharma	FDA approved for peripheral T cell lymphoma
	Panobinostat (Farydak)	Small molecule	Secura Bio	FDA accelerated approval for peripheral T cell lymphoma withdrawn
	Entinostat	Small molecule	Syndax	Clinical development paused due to lack of Phase 3 efficacy
	Tucidinostat (Chidamide, Epidaza or Hiyasta)	Small molecule	Chipscreen Biosciences	CFDA approved for peripheral T cell lymphoma; PMDA approved for adult T cell leukemia-lymphoma
Histone methyltransferase inhibitors	Pinometostat	Small molecule	Epizyme (Ipsen)	Clinical development discontinued due to lack of efficacy
	Tazemetostat (Tazverik)	Small molecule	Epizyme (Ipsen)	FDA approved for relapsed/refractory follicular lymphoma and epithelioid sarcoma
	GSK3326595	Small molecule	GlaxoSmith Kline	Clinical development paused
Lysine demethylase inhibitors	Tranylcypromine	Small molecule	Various	FDA approved for depression
	Ladademstat (ORY-1001)	Small molecule	Oryzion Genomics	In clinical development for multiple tumor types
	GSK2879552	Small molecule	GlaxoSmith Kline	Clinical development discontinued due to unfavorable risk/benefit to patients
Bromodomain inhibitors	Molibresib	Small molecule	GlaxoSmithKline	Clinical development discontinued
	Pelabresib (CPI-06160)	Small molecule	Constellation Pharmaceuticals (MorphoSys)	Clinical development ongoing in myelofibrosis (Phase 3)
	Apabetalone (RVX-208)	Small molecule	Resverlogix	Clinical development ongoing in cardiovascular, infectious disease (COVID-19), and renal disease (Phase 3)
IDH inhibitor	Ivosidenib (Tibsovo)	Small molecule	Servier	FDA approved for the treatment of acute myeloid leukemia and cholangiocarcinoma
	Enasidenib (Idhifa)	Small molecule	Bristol Myers Squibb/Servier	Relapsed/refractory acute myeloid leukemia
Precision epigenomic modulators	OTX-2002	Epigenomic programming	Omega Therapeutics	IND cleared by FDA; clinical trial to begin in 2H2022
	ST-502	Zinc finger protein transcription factor	Sangamo Therapeutics	Preclinical development ongoing
	EPIC-321	Epigenetic engineering	Epic Bio	Preclinical development ongoing

There has also been limited success in applications of epigenetics outside of oncology. Although there is strong evidence that epigenetic dysregulation plays a role in other areas like autoimmunity and hemoglobinopathies, there has been minimal efficacy leveraging available compounds. Well-known HDAC inhibitors like butyrate have shown some proof-of-concept efficacy in certain settings, like sickle cell disease and beta-thalassemia [8], but not enough to outweigh the challenges of tolerability and dosing. Advances in the field that can improve specificity and therapeutic index would ideally help expand the application of epigenetic therapies to a broader range of indications.

### Historical overview of epigenetic drugs and science

**Fig. 2:**
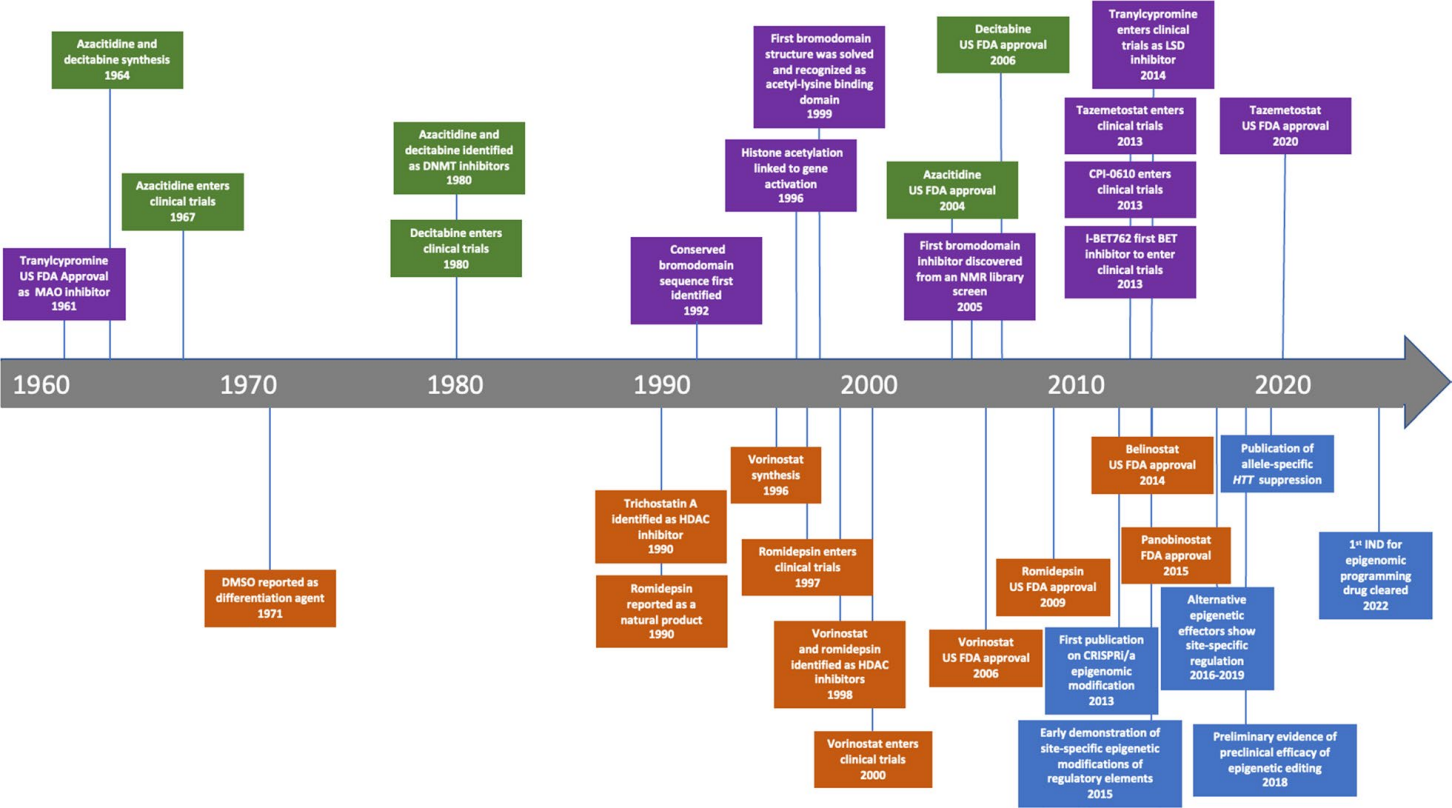
The evolution of epigenetic and epigenomic therapies. The first epigenetic therapeutics were first discovered in the 1960s but the first approval did not come until the early 2000s. Development in the field has accelerated markedly over the past 20 years, with epigenomic programming being the most recent advance. Green = DNMT inhibitor milestones; orange = HDAC inhibitor milestones; purple = 3rd generation epigenetic therapeutic milestones; blue = epigenomic programming milestones

Following the elucidation of the DNA double helix structure, epigenetic markers, DNA methylation, and histone modifications were soon identified [1]. One key advance came in 1974, with the observation that DNA was packaged into nucleosomes, the fundamental subunits of chromatin containing DNA wound around histones [9]. Other discoveries including modification of histone amino-terminal tails and histone acetylation in the 1990s expanded our understanding of how chromatin and other associated proteins ultimately alter gene expression [1, 8–13]. In the past 2 decades, there has been a surge of research activity in the area of histone modifications and the enzymes that make or remove epigenetic marks on DNA and histones (Fig. 2) [1]. Concomitantly, an explosion of research in non-epigenetic modalities for controlling gene expression has occurred. Table 2 summarizes these approaches with their associated strengths and shortcomings.

**Table 2:** Non-epigenetic approaches to modulating gene expression

Modality	Strengths	Weaknesses	Current status	Leading developer(s)	Best applications
Gene/Base/Prime Editing	High specificity Durable/permanent changes Can eliminate pathogenic gene expression and restore/augment expression	Off-target effects (incl DSBs) Limited options for delivery Redosability not possible at this time	1st gen approaches in POC clinical trials 2nd gen approaches in preclinical or early clinical dev	Editas Medicines CRISPR Therapeutics Intellia Therapeutics Beam Therapeutics Prime Medicines	Monogenic diseases with LOF mutations Oncology (ex vivo)
siRNA	Targeted (rational) design Redosable	Only reduces gene Expression Effect is short-lived Knockdown may be incomplete Off-target effects	5 Approved products (US) Many additional efficacy studies in progress	Alnylam Dicerna (Novo Nordisk) Arrowhead Pharmaceuticals	Liver/metabolic disease Infectious disease Rare disease with pathogenic overexpression
ASOs	Multiple MOAs	Less stable than siRNA Durability challenges Low potency Off-target effects	Multiple approved products (US)	lonis Pharmaceuticals Sarepta Therapeutics	Diseases caused by proteins with repeats Liver/metabolic disease Rare disease with pathogenic overexpression
Gene (replacement) therapy	Direct, precise upregulation	Limited options for delivery Redosability not possible at this time	2 approved products (US) Many additional pivotal studies in progress	Spark Therapeutics (Roche) AveXis (Novartis) BioMarin Pharmaceuticals Sangamo Therapeutics	Monogenic loss of function
Cell therapy	Clear connection to disease (known cell type, known modification)	Need appropriate cell type	Multiple approved products but limited to oncology	Novartis Gilead Sciences	Oncology (CAR-expressing cells)
Protein degraders	High tissue selectivity Multiple routes of delivery Well-understood chemistry and manufacturing	Only downregulation/protein reduction Potential for off-target effects	Early to mid-stage clinical development POC is emerging	Arvinas Monte Rosa Therapeutics	Oncology Neuroscience Immunology
Condensates	Differentiated MOA Novel targets	Emerging	Preclinical	Dewpoint Therapeutics Faze Medicines	Oncology Neuroscience/neuro-degeneration

### 1st generation epigenetic drugs

#### DNA methyltransferase (DNMT) inhibitors 

DNA methyltransferases are a class of cytosine methylases that play a key role in epigenetic regulation by depositing marks on the DNA itself. DNA methylation is important in the etiology of cancer as it epigenetically regulates the expression (or lack thereof) of cancer-related genes [14]. In 1980 [15], it was found that structural analogues of nucleo(s)tides could inhibit DNA methylation. Modifications to cytidine led to 5-azacitidine [14, 16] and decitabine [14, 17, 18]. Early work on these compounds yielded promising results in acute myeloid leukemia (AML), but the US applications for marketing authorization were not approved due to toxicity concerns [19, 20]. Subsequent studies were conducted in myelodysplastic syndrome (MDS) using lower doses, leading to FDA approvals for Bristol Myers Squibb [21, 22]. These compounds are also approved in the US to treat chronic myelomonocytic leukemia (CMML) and AML (despite the original FDA rejection), with additional label expansions occurring as recently as 2022 for juvenile myelomonocytic leukemia [21, 23]. Despite current use, the safety profile of these treatments can be difficult to manage and limits their clinical utility. In a recent Phase 3 trial of azacitidine in AML, > 20% of patients experienced Grade 3/4 thrombocytopenia and > 40% experienced Grade 3/4 neutropenia.

Various other nucleoside analogs have also demonstrated DNA hypomethylation activity, but have stalled in development due to low biological activity and/or high levels of toxicity, impacting organs like the liver and heart [16, 24–26]. Studies of derivatives of azacitosine and others remain in early development in various cancers, but are unlikely to represent a significant advance [27]. These early DNMTs provided insights into epigenetic mechanisms and applications in clinical practice while setting the stage for the development of more refined and effective molecules in this class.

#### Histone deacetylase (HDAC) inhibitors

Histone deacetylases are enzymes that remove acetyl marks from lysine residues on histones, allowing chromatin to be wound more tightly, reducing accessibility for transcription. The first epigenetic drugs approved in this class were vorinostat and romidepsin [28]. These agents were discovered through phenotypic observations without an a priori understanding of their mechanism of action as HDACs. Compared with DNMTs, HDACs currently occupy a narrower therapeutic niche [28, 29]. Vorinostat (suberoylanilide hydroxamic acid, SAHA), a pan-HDAC inhibitor developed by Merck [28, 30, 31], proved effective in early studies of several types of cancer. By following the strongest positive data, vorinostat ultimately received FDA approval for cutaneous T cell lymphoma (CTCL) in 2006, supporting the idea that cells of the hematopoietic lineage are most amenable to these broad small molecule epigenetic inhibitors [32, 33]. Potential vorinostat clinical applications extend to treatment of both neurological disorders and reactivation of latent viral infections to increase the efficacy of other antivirals, although additional studies are ongoing [5]. Romidepsin was identified using high-throughput screening studies [28]. Derived from a bacterium, it possessed a unique structure relative to HDAC inhibitors known at the time [34]. It was approved by the FDA in 2009 for the treatment of CTCL [35]. Unlike vorinostat, romidepsin exhibits selectivity between Class I HDACs and other isoforms [28].

Carboxylic acid is another zinc-binding motif that has been studied for its HDAC inhibiting properties [28]. The sodium salt of butyric acid was the first compound shown to inhibit histone deacetylation [36]. Due to rapid excretion, however, and modest clinical activity to date across rare diseases, epilepsy, and cancer, carboxylic acid HDAC inhibitors continue to serve predominantly as research tools. [28, 37]

### 2nd generation epigenetic drugs

#### Second-generation DNMT inhibitors

Assays for DNMT and HDAC activity were available by the early 1990s [28]. Given the limitations of azacitidine and decitabine, several new drugs were developed leveraging these new experimental capabilities. Second-generation DNMTs employ the bi-substrate strategy where a methyl group donor and cytosine are linked together resulting in some of the most potent DNMTi compounds available [28]. These agents can reactivate genes through promoter demethylation in cancerous cells [38]. To date, several of these compounds like guadecitabine (SGI-110), a degradation-resistant hypomethylating CpG dinucleotide mimic, and fluorocyclopentenylcytosine (RX-3117), an oral cytidine analog, have been tested in a range of cancers but none have been approved by the FDA for clinical use due to limited efficacy [4, 39–42]. Other non-nucleoside small molecule DNMTi have been used as preclinical tools and are being evaluated for clinical utility in neoplastic disease [43, 44].

#### Second-generation HDAC inhibitors 

With the second-generation HDACs, applications have broadened to include non-hematological cancers [28]. These molecules tend to have limited efficacy as single agents but have demonstrated clinical utility in combination therapy. Given the efficacy seen with vorinostat, numerous synthetic analogues were developed, leading to the identification of belinostat, which was approved by the FDA in 2014 for the treatment of peripheral T cell lymphoma (PTCL) [28, 32, 45]. Panobinostat gained accelerated approval in combination with dexamethasone and bortezomib from the FDA in 2015 for relapsed or refractory multiple myeloma [32, 46, 47]; however, the approval was withdrawn in 2019. As with the first-generation HDACis, the pharmacokinetic profile of these drugs is not ideal and they can cause off-target effects due to non-selective metal binding [28].

Another successful structural class of compounds are the benzamides, which demonstrate selectivity toward Class I HDACs [28]. One example, entinostat, has been evaluated in clinical trials for multiple solid tumors in combination with hormone therapy and immune checkpoint therapy; however, a lack of robust efficacy data has stalled development [48]. Tucidinostat, a benzamide containing an alkenyl linker, inhibits Class 1 HDACs 1, 2, 3, and class II HDACs and is the first HDACi developed wholly in China, receiving approval from the Chinese FDA in 2015 [32, 49].

### 3rd generation epigenetic drugs

With multiple DNMT and HDAC inhibitors approved for clinical use, the fundamental hypothesis that epigenetics can be harnessed for therapeutic use has been borne out [28]. With improvements in the understanding of epigenetics, though, and the desire to improve the therapeutic window and safety profile of these therapies, efforts have expanded to identify new drugs that target other readers, writers, and erasers.

The third wave of epigenetic drug discovery has identified three new targets: lysine histone methyltransferases (KMTs), lysine demethylases (KDMs), and bromodomain inhibitors [28]. Agents targeting these epigenetic effectors have quickly advanced to clinical trials and regulatory approvals are anticipated in the near future. Unlike the earlier generations, where discovery was serendipitous and the epigenetic effect was unknown, many of these more recent compounds have been identified using prospective knowledge of their mechanisms of action.

#### Histone methyltransferase inhibitors

Histone methyltransferases, either KMTs or protein arginine methyltransferases (PRMTs), post-translationally add between one and three methyl groups to lysine or arginine residues on histone proteins, which can have a range of important effects [28]. Depending on the specific lysine residue being methylated, it can silence or activate gene transcription [50]. Pinometostat (EPZ-5676), developed by Epizyme, was the first KMT inhibitor studied for the treatment of leukemia [51]. Efficacy was, as with previous generations of epigenetic therapies, modest although tolerability was somewhat improved; however, there was a risk of increased infections observed with this agent. Subsequent targeting of the KMT enzyme EZH2 with tazemetostat yielded success for Epizyme in epithelioid sarcoma and follicular lymphoma with FDA approvals in 2020 [52]. Studies in other heme malignancies including diffuse large B-cell lymphoma (DLBCL) are ongoing [32, 53–55]. The first PRMT inhibitor to undergo evaluation in clinical trials was GSK3326595, targeting PRMT5 [56]. Other PRMT inhibitors for PRMT1 and 5 are in clinical trials for the treatment of solid tumors, non-Hodgkin lymphoma, MDS, and DLBCL [57, 58].

#### Lysine demethylase inhibitors

Lysine demethylases reverse lysine methylation on either DNA or histone proteins (among other molecules) which can alter either the transcription of genes at the promoter or via changes in chromatin state. One family of KDMs, lysine-specific demethylases (LSDs) are homologous to monoamine oxidases in their mechanism; thus, MAOIs have been repurposed as epigenetic therapies [59]. Tranylcypromine is an MAOI originally approved in 1961 as an antidepressant but is now in clinical trials as a potential therapy for AML and MDS [31, 60–62]. ORY-1001 and GSK2879552, LSD inhibitors created to improve tranylcypromine’s modest activity and reduce off-target effects, are being tested in Phase I/II trials [32, 63].

#### Bromodomain inhibitors

Bromodomain proteins play a critical role in transcriptional regulation acting via histone acetylation, chromatin remodeling, and recruitment of other transcriptional machinery. Bromodomains are epigenetic readers with the BET (bromo and extra terminal) family garnering the most attention in drug discovery efforts [64]. One of the early compounds, molibresib, has undergone study in Phase I/II clinical trials for NUT midline carcinoma and castration-resistant prostate cancer with some evidence of clinical activity [6, 65, 66]. Other BET inhibitors in clinical development include pelabresib (CPI-0610) being tested in a Phase 3 study of myelofibrosis [67–69] and apabetalone (RVX-208), which is in late-stage development for cardiovascular disease and chronic kidney disease with promising results to date in the non-oncology setting [70].

Although it is well accepted that epigenetic interventions should be feasible therapeutic options outside of oncology, previous small molecule approaches have yielded suboptimal results thus far due to toxicity and off-target effects. With more recent advances, however, we believe epigenetic therapies are poised to become an important component of patient care in the near future (Table 1) [1].

### Potential alternative small molecule strategies

While monotherapy approaches have not produced results as strong as the field expected, there may be promise in combination studies, particularly in solid tumors. Given that tumors often accumulate multiple defects (both mutations and epigenetic dysregulations) as cancers grow and metastasize, combining therapies to target more than one at a time is an enticing hypothesis. As reviewed in Morel et al. [71], the epigenetic effectors targeted by the small molecules detailed above may synergize with a wide array of other therapeutic molecules, including chemotherapies, hormone therapies, and immunotherapy, as a way to either increase overall efficacy or, perhaps more importantly, to overcome acquired resistance.

Some of the more promising combinations have been HDAC inhibition combined with aromatase inhibition in HR^+^ER^−^ breast cancer, HDAC inhibition plus EGFR inhibition in non-small cell lung cancer, and HDAC inhibitors with checkpoint inhibitor treatment in colorectal cancer. In some cases, like that of entinostat plus exemestane, promising Phase II results in breast cancer did not translate into approvable Phase III results [72]; however, a large number of combinatorial trials are still ongoing and may yield more actionable results. Some include pairs of epigenetic therapies, which may be able to better offset defects in tumor cells than monotherapy approaches.

While there is promise to this multi-hit approach, both from preclinical and in early clinical studies, many of the limitations of current epigenetic monotherapies have still arisen. For example, toxicity profiles in combination settings have led to high rates of discontinuations and/or study terminations without a significant boost in survival or tumor shrinkage. Additionally, off-target effects of one epigenetic therapy may be compounded by epi/epi combos, which could offset the benefits. Other tactics may be required to make progress therapeutically harnessing epigenetics.

### Specificity of targeting

While epigenetic therapies have had modest success in oncology, their overall impact has been significantly less than many had hoped and certainly far less than their full therapeutic potential across disease areas. One of the key limitations of these early epigenetic modulators has been specificity. By targeting key effectors in the epigenetic network, one can impact diseased cells, but without a constraint on the sites to which they are delivered (either tissues, cell types, or genomic loci), it is impossible to narrow the impact across all genes and prevent healthy cells from being similarly affected. As such, the levels of toxicity seen with existing classes of inhibitors have restricted their use.

In order to deliver on the broader promise of epigenetics and epigenomics, this issue of molecular specificity must be resolved. The newest era of epigenomic therapies addresses this challenge head-on. Rather than targeting endogenous effector proteins directly, these approaches leverage sequence-specific DNA binding domains (DBD) to direct epigenetic changes to a precise genomic locus or loci. Effector proteins linked to these DBDs allow for exquisite targeting of activity, whether that be writing, modifying, or erasing marks. By leveraging this high degree of sequence specificity, this “epigenomic programming” approach aims to dramatically reduce off-target influences and increase the tolerability and applicability of epigenetic therapies.

The first demonstration of site-specific targeting of epigenetic modifications was published in 2013 by Gilbert et al. [11]. In this in vitro study, guide RNAs (gRNA) were used to target non-editing deactivated Cas protein (dCas) fusions of transcriptional repressors or activators to the promoters of exogenous reporter genes. The effectors were found to be precise and specific, limiting epigenetic changes to the sites encoded by the guides. Similar results were found with endogenous eukaryotic genes. Importantly, the authors were also able to measure significant downstream changes in target gene expression that corresponded with the known activity of the epigenetic effectors but not the DBD (here, dCas) alone.

This proof-of-concept observation was subsequently expanded upon, with additional studies exploring the use of similar systems with alternative effector proteins [15, 19, 49, 73, 74] or to induce histone modifications [75] and changes in activity of regulatory elements [14]. Additionally, several studies have demonstrated that these tools can be used to alter DNA configurations, including conserved loop structures like insulated genomic domains (IGDs), by disrupting or exposing their anchoring CTCF sites to disrupt or restore gene expression [16, 18, 49]. These studies and others confirm the efficacy and utility of site-specific epigenetic changes. The marks conferred by the constructs used in these studies were also confirmed to be long-lasting and in some cases heritable [17], as with endogenous epigenetic marks, and to be capable of inducing gene expression changes via epigenetic reprogramming that resulted in therapeutically relevant improvements in animal models of disease [76]. Together, this body of evidence positioned the field well to begin translating this novel approach into potential therapies.

### Precision genomic control needed

The past several years have seen a number of companies emerge that have taken up the mantle of developing epigenomic technologies into therapies that could represent truly meaningful therapeutic options for patients. Newer epigenetic medicines are targeted to work pre-transcriptionally with high specificity and control over the level of gene expression for a specified period.

Precision and specificity can be conferred utilizing several different DBDs. One commonly used research tool for exploring the potential of these approaches rapidly has been dCas. Here, the enzymatic activity of the Cas protein, made famous by CRISPR gene editing discoveries, has been ablated, allowing this protein to act as a chaperone for an epigenetic effector, directed to the appropriate locus by a separate gRNA. Much of the early literature in the field leverages this technology for proof-of-concept gene activation or silencing.

While this dCas system has been embraced as both a research tool and an emerging therapeutic option, other strategies for targeting use zinc finger proteins (ZFs) or transcription activator-like effectors (TALEs) that encode sequence-specific motifs that directly bind to the DNA without a need for Watson–Crick pairing or the use of separate guide RNA and can be directly linked to effector proteins. These single-component therapeutics, which may have advantages and efficiencies for dosing or multiplexing different effectors to discrete loci in a single therapeutic, are currently under development.

Additional specificity of epigenetic programming relative to small molecule approaches comes from specialized delivery methodologies. Most of these therapeutics are delivered via cell- or tissue-specific vectors. Many drug developers are delivering DBD-effector fusions encoded as DNA and encapsulated in viral vectors like adeno-associated viruses (AAVs). AAVs have distinct tropisms for certain cells/tissues based on serotype, providing greater control compared to systemic delivery of small molecules, which generally have indiscriminate biodistribution. AAV-delivered approaches tend to generate long-lived or even permanent expression systems; however, in the context of epigenomic therapies, these suffer from concerns over redosability due to pre-existing or induced immunogenicity to the viral capsid and the inability to withdraw or negate the activity of the effectors if any untoward effects are observed clinically. Further, the payload capacity of the AAV genome is limited, making it challenging to encode large sequences like dCas and linked effector domains plus guide RNAs in a single viral vector.

An alternative option to viral vectors being employed increasingly is lipid nanoparticles (LNPs) [12, 13]. These vehicles allow for similar cell/tissue-restricted delivery based on the composition of lipids utilized but avoid several of the limitations of AAVs. LNPs are redosable as a result of generally favorable tolerability and limited immunogenicity, providing opportunities for long-term administration across therapeutic areas like oncology, inflammation, and chronic disease. Additionally, based on LNP structure and mRNA properties, there are fewer restrictions around payload capacity and the size of therapeutic constructs encoded as mRNA. While there are also certain challenges to working with LNPs, they provide significant optionality in the epigenetics space.

One of the unique advantages to working with mRNA/LNPs is the ability to decouple the pharmacokinetics (PK) and pharmacodynamics (PD) of these novel therapeutics which is not possible with earlier generations of small molecule epigenetic modulators or viral delivery. mRNA-encoded approaches using LNPs are transient expression systems. While the expressed therapeutic proteins themselves typically persist in vivo on the order of days, there is robust evidence that the epigenetic changes imparted can be heritable and the phenotypic effects on expression can last on the order of weeks or months; the durability can be specified depending on the nature of the intervention (e.g., effector selection) and the requirements of the specific indication and gene target. As such, the epigenomic medicines delivered in this way can provide major advantages, including meaningful reduction in dosing frequency and a potential significant improvement in safety profile, which can help expand the clinical utility of these drugs into a wider array of diseases.

In addition to increased specificity and durability, novel epigenomic therapies have the ability to tune gene expression rather than induce a binary on or off result. Certain genes, including many notable oncogenes, are required for normal function of cells but can be pathogenic if overexpressed. By intervening pre-transcriptionally with the appropriate epigenetic effectors, it is possible to modulate gene expression to a physiologically normal level without completely ablating expression, which could have other negative effects. The same holds true for targets requiring upregulation; restoring homeostatic expression rather than supraphysiological levels may be safer or more tolerable in the long term.

A final feature that differentiates precision approaches from other genetic medicine strategies and earlier generations of epigenetic therapies is the ability to precisely multiplex targets to enhance efficacy. Multiplexing can take different forms, including the use of more than one effector targeted to a given region to synergistically drive multiple epigenetic changes or the epigenetic modulation of multiple genes that contribute to pathology in the same disease state to obtain improved efficacy. In both cases, this multiplexing utilizing, for example, multi-cistronic mRNA encoding multiple therapeutic proteins, is distinct from the off-target effects of small molecule epigenetic therapies which had broadly detrimental impacts. Being able to address genes in the context of their natural DNA structure, for example in IGDs, allows for epigenetic regulation of not only promoters but other regulatory elements like enhancers, repressors, and CTCF sites or long non-coding RNAs, which can have profound, tunable, and durable effects. Directing epigenetic effectors to multiple genes via one therapeutic can also overcome some of the challenges to treating complex diseases where it is not feasible to utilize multiple small molecules or biologics simultaneously due to safety or tolerability issues. The therapeutic potential unlocked by these advances in precision epigenomic programming is truly remarkable.

### Entry into the clinic for epigenomic programming

The application of epigenomic technologies spans a wide range of therapeutic areas, in agreement with the broad reach of epigenetics. Development is underway in oncology, cellular regeneration, autoimmune disease, ex vivo cell therapy, rare genetic disorders, and neuroscience including neurodegenerative diseases. A 2019 publication by Zeitler et al. [20] details the allele-specific repression of mutant *HTT* as a strategy to combat Huntington’s disease. By targeting a zinc finger protein-KRAB fusion to the amplified CAG repeats, the effector was able to repress transcription selectively with therapeutic benefit in both behavioral and histological measures in a murine model. In a 2021 presentation, both in vitro and in vivo data also provided evidence to support therapeutic downregulation of a-synuclein in a murine model utilizing a zinc finger protein linked to a KRAB repressor delivered in an AAV vector.

In oncology, preclinical work in epigenomic programming has demonstrated significant progress on targeting cMYC, a key oncogene historically considered undruggable. Leveraging IGD biology, epigenomic controllers targeted the IGD containing MYC were able to robustly downregulate expression of the gene across both hepatocellular carcinoma (HCC) and non-small cell lung cancer (NSCLC) cell lines. This decrease in MYC expression led to a concomitant decrease in tumor cell viability in vitro, with the associated epigenetic marks detectable at the sites of interest 2 weeks post-administration. Importantly, normal primary cells from both the liver and lung did not show the same increase in cell death, supporting the more targeted effect of targeted epigenomic therapies over other epigenetic drugs. In vivo*,* MYC-targeting via epigenomic programming led to a decrease in tumor size in multiple murine xenograft models. Together, these early publications and presentations are evidence of substantial progress in translational research for epigenomic programming and speak to the longer-term promise in clinical development. The first clinical trial for an epigenomic programming approach is currently ongoing with the first patient dosed in October 2022.

## Conclusions and future directions

While the evolution of the epigenetic therapeutics field has been marked by modest success alongside notable disappointments, the rise of new precision approaches is expected to be a transformational catalyst to unlock its full potential. Similarly, great strides have been made in recent years in furthering our understanding of the causal links between dysregulation of the epigenome, disease processes, and negative outcomes, contributing to greater clarity on the most relevant targets to pursue. We believe that this potent combination of enhanced mechanistic understanding coupled with new and highly refined tools with unprecedented specificity portends a revolution in the ability of epigenomic medicine to deliver on the full breadth of its promise of transformational therapies for patients living with cancer and other chronic life-threatening conditions.

Although clinical development in this space is just beginning, we anticipate rapid progress over the next several years. Success in unlocking targets previously considered undruggable, like MYC, could yield orthogonal strategies for treating patients, either as monotherapies or in conjunction with existing approaches. The ability to integrate new targets into the therapeutic landscape in oncology could be a major advance for patients, particularly those who are not amenable to existing targeted therapies, although it may take some time to understand the ideal patient characteristics and associated biomarkers to best apply these technologies. Similarly, the regenerative potential of the epigenomic programming approach has the potential to address debilitating neurodegenerative diseases as well as other diseases of aging in a way that has not been achievable to date. Targets that are known to be critical in disease biology but have eluded existing technologies represent a meaningful opportunity to improve patient outcomes. Harnessing epigenomic programming could yield practice-changing medicines in many areas if current technologies can be translated into clinical results.

Building on the lessons learned from the earliest clinical efforts and maximizing the new levels of control conferred by improvements in targeting, protein engineering, and delivery of genetic medicines, epigenetics has the potential to deliver on its promise in a significant way for a wide variety of patients in need.

## Data Availability

Data sharing is not applicable to this article as no datasets were generated or analyzed during the current study.
